# Prioritizing tasks in software development: A systematic literature review

**DOI:** 10.1371/journal.pone.0283838

**Published:** 2023-04-06

**Authors:** Yegor Bugayenko, Ayomide Bakare, Arina Cheverda, Mirko Farina, Artem Kruglov, Yaroslav Plaksin, Witold Pedrycz, Giancarlo Succi

**Affiliations:** 1 Huawei, Moscow, Russia; 2 Institute of Software Development and Engineering, Innopolis University, Innopolis, Russia; 3 Institute of Human and Social Sciences, Innopolis University, Innopolis, Russia; 4 Department of Electrical and Computer Engineering, University of Alberta, Edmonton, Canada; 5 Department of Computer Science and Engineering, University of Bologna, Bologna, Italy; Firat Universitesi, TURKEY

## Abstract

Task prioritization is one of the most researched areas in software development. Given the huge number of papers written on the topic, it might be challenging for IT practitioners–software developers, and IT project managers–to find the most appropriate tools or methods developed to date to deal with this important issue. The main goal of this work is therefore to review the current state of research and practice on task prioritization in the Software Engineering domain and to individuate the most effective ranking tools and techniques used in the industry. For this purpose, we conducted a systematic literature review guided and inspired by the Preferred Reporting Items for Systematic Reviews and Meta-Analyses, otherwise known as the PRISMA statement. Based on our analysis, we can make a number of important observations for the field. Firstly, we found that most of the task prioritization approaches developed to date involve a specific type of prioritization strategy—*bug prioritization*. Secondly, the most recent works we review investigate task prioritization in terms of “pull request prioritization” and “issue prioritization,” (and we speculate that the number of such works will significantly increase due to the explosion of version control and issue management software systems). Thirdly, we remark that the most frequently used metrics for measuring the quality of a prioritization model are *f-score*, *precision*, *recall*, and *accuracy*.

## Introduction

In software development, the vast majority of tasks do not have mandatory dependencies and it is up to the project manager to decide which task should be completed first. The proper continuous prioritization of tasks (known as *backlog refinement* in agile terminology) becomes a critical success factor for any software development project, as it guarantees that the company’s crucial goals are in focus and can be met [1].

What is a task, though? The term “task” in software engineering refers to the smallest unit of work subject to management accountability that needs to be completed as part of a software development project [2]. So, in the context of software development, the term task is an umbrella term that encompasses concepts, such as “pull request” and “issue,” commonly found in GitHub/GitLab integration (so development areas) [3], or to ideas, such as “bug,” “feature,” “improvement,” commonly used in task management. Although these concepts and ideas are considered conceptually independent, they often overlap in practice.

In an attempt to optimize the process and practice of task prioritization, researchers approached the problem from a bug-fixing perspective; that is, in terms of selecting the most appropriate developer for the given task [4]. Cubranic and Murphy were among the first to analyze the problem of task prioritization in terms of Machine Learning (ML); namely as a classification problem [5]. The datasets provided in their research, Eclipse (see https://bugs.eclipse.org/bugs/) and Mozilla (see http://www.mozilla.org/projects/bugzilla), have become “de facto” the standard for training and testing ML models for this problem domain.

However, it is worth noting, that other researchers developed alternative methods and approaches to improve the process of prioritizing and assigning bug fixes. For example, Zimmermann et al. [6] provided a series of recommendations for formulating and better classifying bug reports, while Anvik et al. [7] proposed an effective strategy for developers selection. Panjer [8] formulated a method capable of predicting bugs’ lifetime and Wang et al. [9] suggested a new technique for identifying bug duplicates.

Menzies and Marcus [10] adopted another conceptual framework for dealing with the problem of task prioritization and proposed a solution based on the prediction of the severity of bug reports. Their work formed the conceptual palette necessary for the development of further research on bug priorities prediction, such as the works by Sharma et al. [11] and Tian et al. [12].

The importance, urgency, and significance of this problem for the Software Engineering community is also attested by the recent publication of several surveys, such as [13–16]. Among them, the work of Gousios et al. showed that the issue of task prioritization is particularly sensitive for development teams that follow a pull-based development model [16–18].

The considerations made above clearly demonstrate that task prioritization has become an active research topic in software engineering. On the one hand, its growth signals a positive trend: the more people get involved in the discussion of these issues, the more ideas are generated and accumulated in the scientific community. On the other hand, though, wide participation poses potentially insurmountable challenges for researchers and developers in terms of understanding the current state and capabilities of the field. Therefore, we believe that a comprehensive systematic literature review (SLR) carried out on this topic is going to be highly beneficial for researchers, project managers, developers, scrum masters, and other industry practitioners.

### Research problem and objectives

Taking this important observation as a starting point this work reviews how the IT industry addresses the problem of task prioritization and attempts to produce a state-of-the-art summary of tools and techniques used for this purpose. Although we do not limit our work to specific methods, we expect to mostly gather Machine Learning (ML)-based approaches. This is because of the recent successes of ML in software engineering and computer science [19, 20].

The objectives of the SLR are therefore to:

- present our readership (mostly IT practitioners) with newly-developed techniques for ranking tasks that they can reliably use in their work,
- develop new strategies in ranking and prioritizing tasks, thus filling current gaps in the relevant literature and
- identify possible directions for future research.

The scientific contribution of this paper includes structured information on task prioritization, a survey of existing tools and approaches, methods, and metrics, as well as some estimates about their effectiveness and reliability.

### Structure of the paper

This paper is organized as follows. Section Related Works provides an overview of current research on task prioritization and a helpful comparison between such research and the focus and scope of our work. Section SLR Protocol Development describes the protocol used in this systematic literature review. Section Results presents the results of our work, while section Discussion contextualizes our findings and section Critical Review of our Research Question provides their critical interpretation. Section Limitations, Threats to Validity, and Review Assessment evaluates the limitations and various other shortcomings potentially affecting our study, while section Conclusion summarizes what we achieved and points out future research directions.

## Related works

There exist a number of studies devoted to requirements prioritization techniques. For example, Achimugu et al. [21] found that the most cited techniques for requirements prioritization include Analytical Hierarchy Process (AHP), Pairwise Comparison, Cost-Value Prioritization, and Cumulative Voting. More recent trends in prioritizing requirements include ML techniques (such as Case Base Ranking and Fuzzy AHP). Bukhsh et al. [22] also identified a trend toward fuzzy logic and machine learning methods. Somohano-Murrieta et al. [23] investigated the most documented techniques with regard to scalability and time consumption problems. Rashdan [24] found evidence of a shift towards computed-assisted/algorithmic methods, while Sufian et al. [25] analyzed factors that influence prioritization and identified commonly used techniques and tools aimed at improving the process. These studies underline the importance and evolution of requirements prioritization techniques, and -at the same time- emphasize the need for real-world evaluations and scalability solutions.

There are also studies aimed at analyzing other aspects of software engineering, which are typically connected with prioritization issues (such as analysis of non-functional requirements, code smells, technical debt, and software bugs). For example, Kaur et al. [26] identified existing techniques for code smell prioritization and introduced different tools for prioritizing code smells (such as Fusion, ConQAT, SpIRIT, JSpIRIT, PMD, Fica, JCodeOdor, and DT-SOA). Alfayez et al. [27] investigated technical debt prioritization and identified a number of important techniques used, which include: Cost-Benefit Analysis, Ranking, Predictive Analytics, Real Options Analysis, Analytic Hierarchy Process, Modern Portfolio Theory, Weighted Sum Model, Business Process Management, Reinforcement Learning, and Software Quality Assessment Based on Lifecycle Expectations (SQALE). However, the researchers concluded that more research is needed to develop technical debt prioritization approaches capable of effectively considering costs, values, and resource constraints. Ijaz et al. [28] looked at non-functional requirements prioritization techniques and found that AI techniques can potentially handle uncertainties in requirements while contributing to overcome the most common limitations characterizing standard approaches (such as AHP). Pasikanti and Kawaf [29] studied the latest trends in software bug prioritization and identified a series of ML techniques (such as Naive Bayes, Support Vector Machines, Random Forest, and Multinational Naive Bayes) that are most commonly used for prioritizing software bugs.

Several SLRs were also conducted to identify the most commonly used techniques for test case selection and prioritization in software testing. For example, Pan et al. [30] found that Supervised Learning, Unsupervised Learning, Reinforcement Learning, and NLP-based methods have been applied to test case prioritization; yet, due to a lack of standard evaluation procedures, the authors couldn’t draw reliable conclusions on their effective performance. Bajaj and Sangwan [31] observed that genetic algorithms bear great potential for solving test case prioritization problems, while nevertheless noting that the design of parameter settings, type of operators, and fitness function significantly affects the quality of the solutions obtained.

Another important area of research focuses on aspects of task assignment and allocation in software development projects. Filho et al. [32] reviewed works on multicriteria models for task assignment in distributed software development projects with a special focus on qualitative decision-making methods. TAMRI emerged as the most efficient and widely used approach, while McDSDS, Global Studio Project, and 24-Hour Development Model received lower scores. Fatima et al. [33] studied the models used for task assignment and scheduling in software projects. The review found that static models are the most widely used for task scheduling, while the Support Vector Machine algorithm is the most widely used for task assignment. Both these papers demonstrated the importance of considering, as crucial for the practice of software management, specific factors (such as personal aspects, team skills, labor cost, geographic issues, and task granularity).

However, the contribution of our SLR is unique and different from that of the above-mentioned studies because: (Table 1):

- Unlike other SLRs, which have focused -as we have seen above- on prioritization techniques for requirements, test cases, bugs, and/or other artifacts of software development; our own review provides a comprehensive coverage of the problem at stake. Crucially, it does so by describing the broad category of “task”, without focusing on a specific type of prioritized item.
- In addition, our research differs from prior studies on task allocation/assignment in several aspects. Firstly, the problem of assignment/allocation involves distributing tasks based on various factors (such as skills, availability, workload, etc), whereas the problem of prioritization focuses on determining which tasks should be completed first. Secondly, prior research has predominantly relied on qualitative analyses of algorithms, methods, and tools for task allocation/assignment, without conducting detailed quantitative analyses of their effectiveness. Our research aims to bridge these important gaps in the literature by conducting a comprehensive quantitative analysis of task prioritization techniques, which are used to determine their effectiveness in different contexts.

**Table 1:** Summary of existing related literature reviews.

Reference	Covered years	Number of studies	Domain	Focus
Achimugu et al. [21]	1996–2013	73	Software requirements	Requirements prioritization techniques
Sufian et al. [25]	2009–2017	33	Software requirements	Requirements prioritization techniques and tools
Bukhsh et al. [22]	2007–2019	102	Software requirements	Requirements prioritization methods and their empirical evaluation
Somohano-Murrieta et al. [23]	2010–2019	35	Software requirements	Requirements prioritization techniques
Rashdan A. [24]	2014–2020	53	Software requirements	Taxonomy and trends in requirements prioritization techniques
Bajaj and Sangwan. [31]	1999–2018	20	Regression testing	Use of genetic algorithms in test case prioritization
Pan et al. [30]	2006–2020	29	Regression testing	Use of ML techniques for test case selection and prioritization
Kaur et al. [26]	till 2020	23	Code smells	Code smell prioritization techniques and tools
Alfayez et al. [27]	1992–2018	23	Approaches and techniques for technical debt	technical debt prioritization
Ijaz et al. [28]	2008–2019	30	Non-functional requirements	Non-functional requirements prioritization methods, including AI, and their validation
Pasikanti and Kawaf [29]	2015–2022	34	Software defects and bugs	Techniques, algorithms and methods of defects or bugs prioritization
Filho et al. [32]	till 2016	21	Task assignment	Qualitative decision-making models for assigning tasks in distributed software development projects
Fatima et al. [33]	2012–2019	23	Task assignment	Techniques and ML algorithms for software project scheduling
This study	2006–2022	83	Task prioritization	ML methods and metrics for task prioritization

## SLR protocol development

SLRs offer a comprehensive analysis of the research conducted in the field while also providing critical, original insights [34]. They are of paramount importance for scientific progress and, for this reason, represent one of the preferred methods used by researchers to investigate the state of the art of a particular research topic [35].

The quality of SLRs can vary greatly and it is important to ensure that an SLR is conducted in a rigorous and systematic manner [36, 37]. Thus, to ensure the comprehensiveness and soundness of our work we followed the PRISMA Statement [38], which is essentially a checklist, conventionally adopted by researchers worldwide, to guide, orient, and inform the development of any SLR. The PRISMA 2020 checklist adopted for this study is included as S1 Table.

Since the Prisma checklist abovementioned is not -strictly speaking- a methodological framework; rather a series of suggestions or -better- recommendations to be implemented for the sound development of any SLR (even beyond computer science), we decided to integrate it and complement it with a more specific methodological framework; the one recently developed by Kitchenham and Charters [39]. This framework was chosen due to its focus on software engineering and because its effectiveness has been amply demonstrated in previous studies [40–42]. We believe that complementing the general indications or recommendations outlined in the PRISMA checklist (which are valid for any field) with a framework specifically designed for research on software engineering is highly beneficial for this study, as it guarantees better accuracy. In addition, since the checklist and the framework partially overlap (despite being also complementary), one can use them to mutually strengthen each other. The stages of the methodological framework adopted in this SLR, are:

1. Specification of the research questions.
2. Development of the review protocol.
3. Formulation of the literature log.
4. Performance of quality assessment.
5. Extraction of Data.
6. Data synthesis.
7. Formulation of the main report.
8. Evaluation of the review and of the report.

### Research questions

The first step in any SLR involves the formulation of a series of research questions that can guide and inform its development.

To formulate the most appropriate research questions, we adopted the Goal Question Metric (GQM) model developed by Basili et al. [43]. This model requires specifying up front the purpose of analysis, the objects and the issues to analyze, as well as the standpoints from which the analysis is performed. The Goal Question Metric model for this work is the following:

**Purpose** Systematic literature review.
**Object** Peer review publications in computer science and software engineering.
**Issue** Approaches for ranking tasks in software development.
**Viewpoint** Software engineers and industry practitioners.

With the GQM model in place, we then formulated the Research Questions (RQs) that characterized this work:

**RQ1** What are the existing approaches for automatic task ranking in software development?
**RQ2** Which methods are used in automatic task ranking models and approaches and how is their effectiveness assessed?
**RQ3** What are the most effective and versatile models for automatic task ranking developed so far?

The motivation for RQ1 is to gain a clear understanding of existing research on the topic. Then, moving from general to more specific tasks, we formulate RQ2 with the intent of finding out which methods for task ranking are currently the most popular in the software development industry and how their effectiveness can be assessed. Further research along these lines leads to RQ3, through which we try to rank such methods in terms of effectiveness, accuracy, fidelity, and reliability. This could help developing new ranking strategies and remedial approaches for the field.

### Literature search process

Following the best practices in the field [42], we selected the following databases for our searches: Google Scholar, Microsoft Academic, ScienceDirect, IEEE Xplore, and ACM digital library.

We then extracted a set of basic keywords, which describe our research questions. The keywords are: *a)* manage, *b)* backlog, *c)* priority, *d)* task, *e)* job, *f)* commit, *g)* bug, *h)* pull request, *i)* issue, *j)* feature, *k)* software, *l)* rank, *m)* distributed software development, *n)* machine learning.

Searches via keywords yielded a very large number of papers. Thus, to screen out irrelevant documents and add focus and precision to our work, we formulated a set of search queries by using Boolean operators, as common in the literature. Upon conducting an initial screening of papers, we discovered that there were more papers focused on prioritizing bug reports than those focused on prioritizing pull requests and issues. Because of this, we decided to model a series of search queries around these themes for better coverage. The list of queries we used for our searches is reported below:

1. ((pull OR merge) AND request) OR Github issue) AND (prioritization or priority OR rank OR order OR ranking OR ordering)
2. (task or bug or defect or feature) AND (prioritization or priority OR rank OR order OR ranking OR ordering)
3. bug severity AND priority AND (machine learning OR neural network)

We performed our searches by using these queries on the selected databases. Table 2 displays the results we obtained.

**Table 2:** Results of the search queries in a number of scientific databases. Acronyms used: GS—Google Scholar, IEEE—IEEE Xplore, MA—Microsoft Academic, SD—ScienceDirect.

Query	GS	MA	SD	IEEE	ACM
1	52400	9106	143702	0	582019
2	20500	10619	1813	2	431329
3	22000	13177	430	10	66381
Total	358900	36842	15118	256	1108517

### Inclusion and exclusion criteria

Next, we specified inclusion (IC) and exclusion (EC) criteria as recommended by Patino and Ferreira [44]. IC and EC help the authors decide which articles found through seminal searches deserve to be considered for further analysis. In this study we used the following IC and EC:

**IC1** The paper is written in English.
**IC2** The paper is peer-reviewed and published by a reputable publisher.
**IC3** The paper was published as early as 2006*.
**IC4** The paper uses ML techniques to deal with backlog systems or tasks/todos.
**IC5** The paper compares different ML models or compares ML models with other learning models.
**EC1** The paper does not satisfy at least one of the ICs.
**EC2** The paper is a duplicate or contains duplicate information.
**EC3** The paper is an editorial, an opinion piece, or an introduction. In general, the paper is excluded if it does not contain any original insight.
**EC4** The paper does not present any type of experimentation or comparison or results.

*Shoham et al. [45] noted that around 2006, there was a pick of interest in ML in the software engineering community. We thus selected this year as the starting point for our systematic review.

### Search results by sources

In this subsection, we offer to our readers a detailed description of the process that led to the inclusion of preliminary selected papers in our final reading log (Table 3).

**Table 3:** Papers selection. The table shows the procedure through which potentially relevant papers were screened out through the adoption of IC and EC criteria. The number of papers included in the final reading log is shown in the column “Selected papers”.

Source	Initial selection	Potentially relevant	Removed papers									Selected papers
			IC1	IC2	IC3	IC4	IC5	EC1	EC2	EC3	EC4	
Google Scholar	400	117	1	5	5	1	-	-	42	4	-	59
Microsoft Academic	400	0	-	-	-	-	-	-	-	-	-	-
ScienceDirect	400	41	-	2	-	6	-	-	24	-	-	9
IEEE Xplore	256	41	-	-	-	-	-	-	29	-	-	12
ACM	400	22	2	-	-	-	-	-	13	4	-	3

We note that we only considered the first 100 results displayed in the relevant databases for each of the four queries we formulated. This is justified by the fact that the databases we used normally sort out results by significance and credibility (e.g., h-index, number of citations, impact factor, etc.) and by the observation that usually no relevant paper is found after the first 100 results.

**Fig 1:**
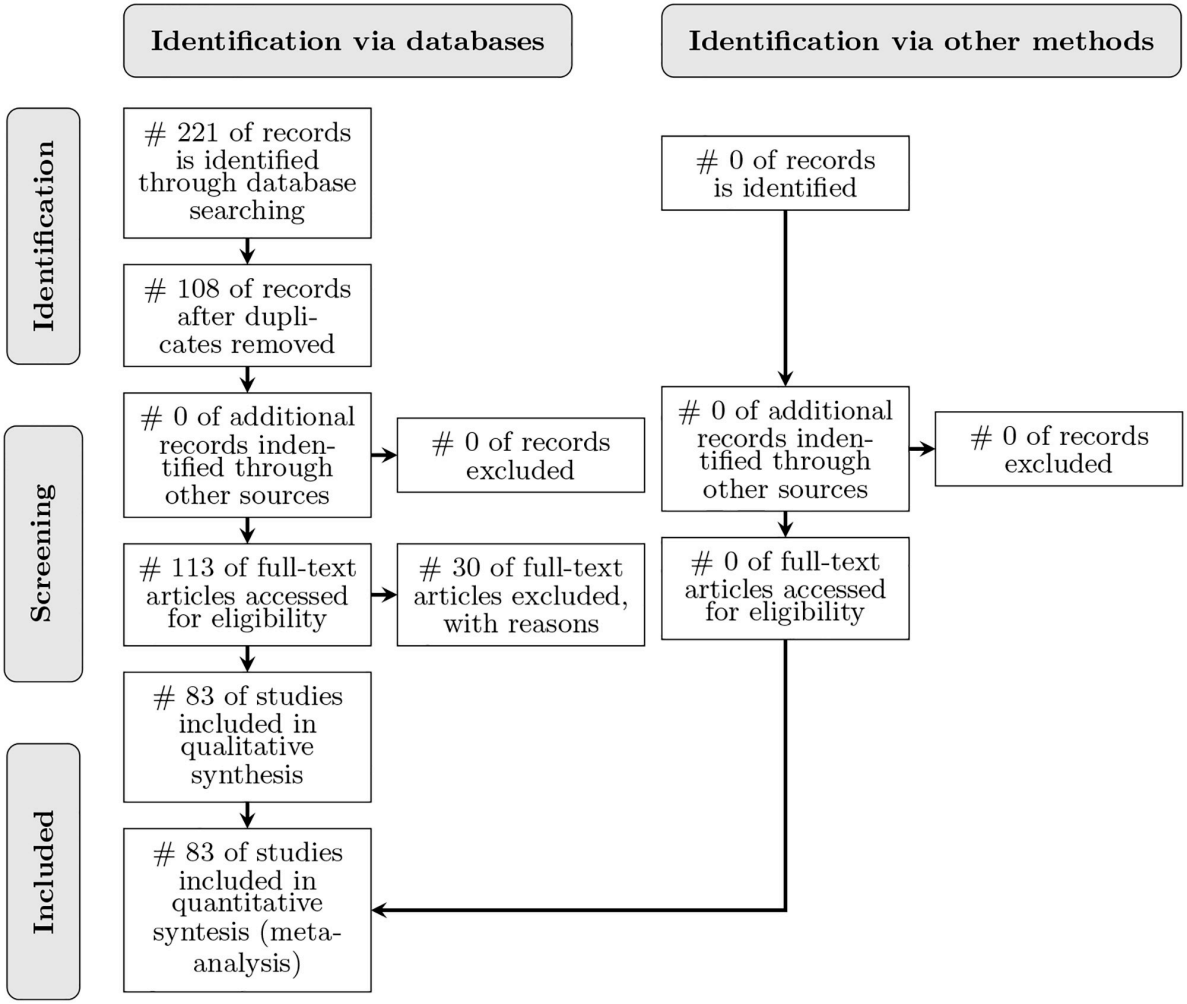
PRISMA flow diagram. It shows the stages of the search process as a flowchart diagram [38].

The PRISMA flow chart diagram shown in Fig 1 represents the process of inclusion/exclusion visually for the reader.

### Quality assessment

To assess the quality of the manuscripts, we defined a set of criteria and applied them to all the papers selected for inclusion in our reading log:

**QA1** Were the objectives and the research questions clearly specified?
**QA2** Were the results evaluated critically and comprehensively?
**QA3** Was the research process transparent and reproducible?
**QA4** Are there comparisons with alternatives?

We then determined whether the papers we selected matched the criteria and—in case—the extent to which they did so. So, we assigned 1 if a paper fully matched the criterion, 0.5 if it partially matched the criterion, and 0 otherwise.

The criteria used for QA1 are:

**Fully matched** The objectives and research questions were explicitly stated.
**Partially matched** The goals of the paper and its research questions were sufficiently clear but could be improved.
**Not matched** No objectives were stated if the research questions were hard to determine, or if they didn’t relate to the research being carried out.

The criteria used for QA2 are:

**Fully matched** The authors of the paper provided a critical, balanced, and fair analysis of their results.
**Partially matched** The results were only partly (sufficiently) scrutinized and a comprehensive critical analysis was missing.
**Not matched** The authors did not evaluate their results.

The criteria used for QA3 are:

**Fully matched** The paper specified the methodology and the technologies used as well as the data gathered.
**Partially matched** Minor details were lacking (for example, a dataset is not readily available).
**Not matched** It was impossible to restore the sequence of actions or if other critical details (such as an algorithm or technologies used) were missing.

The criteria used for QA4 are:

**Fully matched** A comparison with other solutions offered; advantages and limitations clearly stated.
**Partially matched** The comparison was offered, but it was not comprehensively discussed.
**Not matched** No comparison was provided.

**Fig 2:**
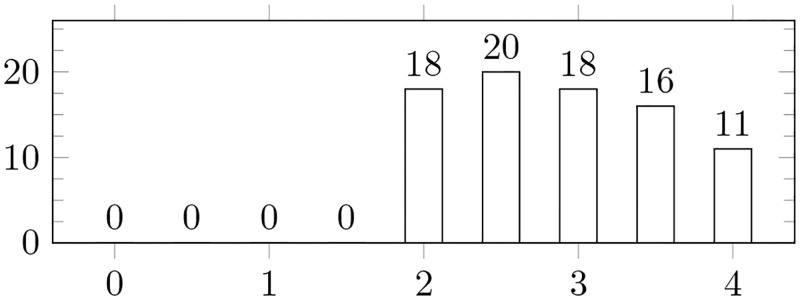
Papers distribution by quality score. Each paper was evaluated on a scale from 0 to 1 as per QA1-QA4. The bars display the number of papers with their respective quality score.

The resulting scores are shown in S2 Table, and their distribution can be found in Fig 2.

There were many high-quality papers among those we selected for inclusion in our final log, which is demonstrated by the scores reported in Fig 2. The average quality score was 2.9 out of 4. This confirms the reliability of the findings on which we based our SLR.

## Results

This section presents the findings gathered from the papers we included in our final reading log. More specifically, in this section, we use a series of statistical tools to cluster and organize the papers we selected in meaningful ways. Such clustering is beneficial for our readers because it provides some background for the conclusions we will draw in subsequent sections.

### Preliminary clustering

We start this process of clustering by summarizing the potential advantages and disadvantages of the databases we used to perform our searches.

**Microsoft Academic** has the advantage of extensive coverage of scientific research, including patents. Its limitation is that some of the papers it lists are not peer-reviewed.
**IEEE Xplore** provides peer-reviewed publications, generally of high quality. Its limitation is that its full functionality requires a subscription, which is pricy.
**ScienceDirect** offers comprehensive coverage with tools for statistical analysis. However, it is beyond a paywall and has limitations for query building.
**ACM** provides comprehensive coverage with a particular emphasis on IT. Its major limitation is that it requires a subscription.
**Google Scholar** is one of the best database aggregators. It provides comprehensive coverage and tools for statistical analysis. However, it includes grey literature and non-peer-reviewed publications.

While not of crucial importance for the development of this work, we notice that such–complementary–information can be useful to ensure the academic integrity and scientific soundness of our approach.

**Fig 3:**
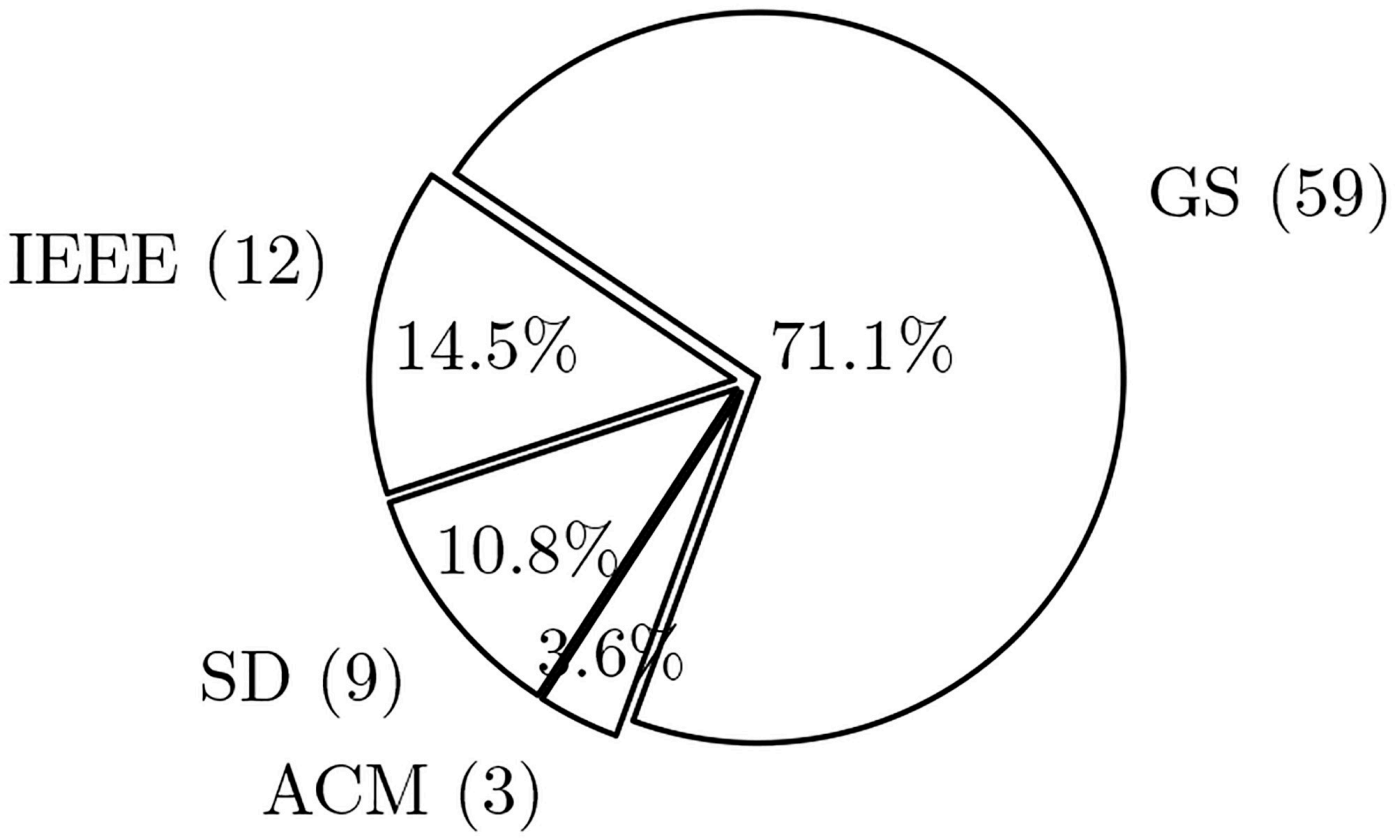
Papers distribution by databases. The pie chart shows the percentage of papers found in the databases we considered in this study. Acronyms used: GS—Google Scholar, IEEE—IEEE Xplore, SD—ScienceDirect. The number of papers is given in brackets.

The distribution of the papers included in the final reading log by databases is presented in Table 3. Fig 3 re-elaborates the information contained in Table 3 in the form of a pie chart, which is probably more appealing for the reader. For convenience, we attributed papers to single repositories (even though some papers could be found across different databases). The attribution was subjective in character and determined by the chronological order of the searches we performed.

**Fig 4:**
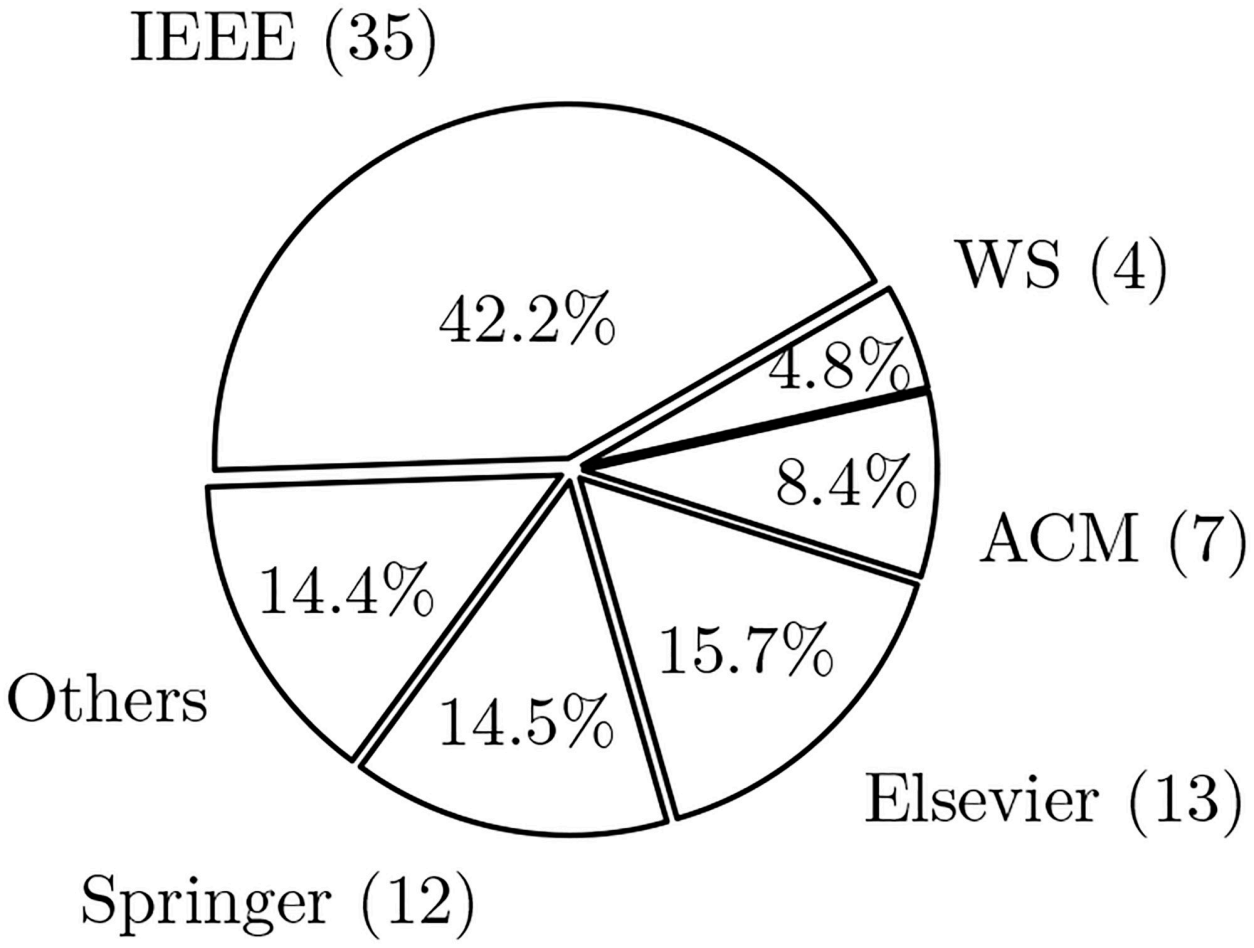
Papers distribution by publishers. The pie chart shows the distribution (in percentages) of the papers we considered in our reading log by publishers. Acronyms used: IEEE—IEEE Xplore, WS—World Scientific.

To give the reader a fuller picture of our results, we added information about the distribution of papers by publisher. This information can be found in Fig 4. We note that the following journals and publishers fall under the label “others,” which accounts for about 14% of selected studies: ASTL (SERSC), CES (hikari), EISEJ, IJACSA, IJARCS, IJCNIS, IJCSE, IJOSSP, JATIT, Sensors (MDPI), and TIIS (KSII).

### Studies classification

In this subsection, we present a series of statistical data that can be used to cluster our findings. Firstly, we identified 2 major topics characterizing the studies we included in our reading log: “Bug prioritization”, “Bug severity prediction”, and 2 minor topics “Issue prioritization”, and “Pull Request prioritization”. It is worth noting that even though bug severity [46] and bug priority [47] are two different theoretical entities (often treated as such even by project managers), a few works [12, 48, 49] demonstrated that severity can sometimes help predict priority. This is why, in this study, we consider not only papers concerned with bug priority but also those related to bug severity. Table 4 shows the distribution of publications across these topics.

**Table 4:** Papers distribution by key topics. The table shows the number (column “Quantity”) of papers devoted to a particular key topic (column “Topic”). Note: 2 papers have content for both topic 1 and 2 distribution.

Topic	Earliest publication date	Latest publication date	Quantity
Bug prioritization	2010	2022	31
Bug severity prediction	2012	2022	43
Issue priority	2014	2020	4
Pull Request priority	2014	2021	6

**Fig 5:**
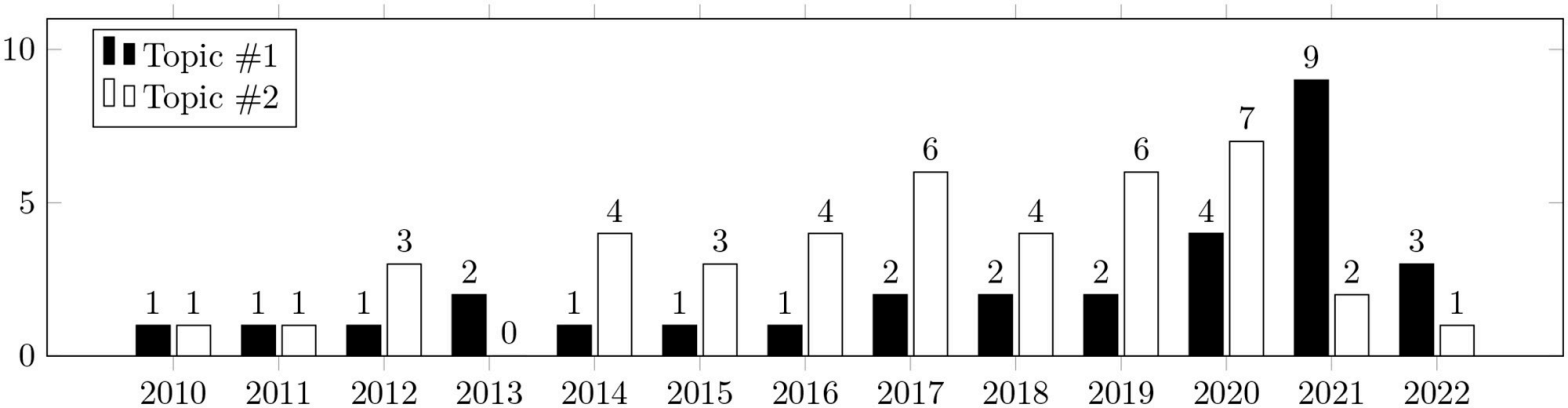
Topics distribution by year of publication. The bars show the number of papers related to the key topic published in a particular year. Black bars show the number of papers related to “bug prioritization”. White bars show the number of papers related to “bug severity and prediction”.

**Fig 6:**
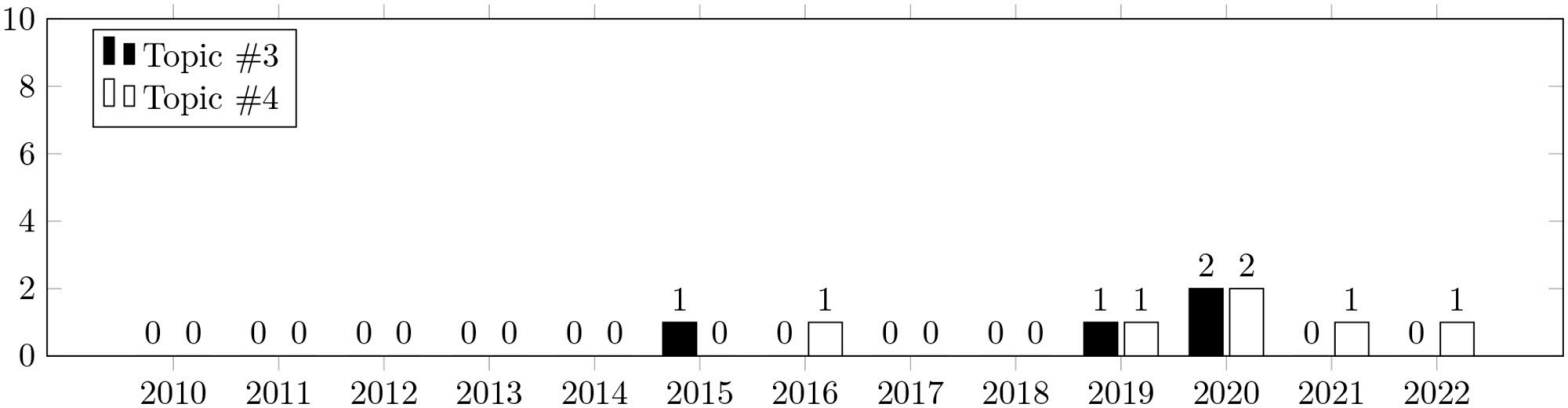
Topics distribution by year of publication. The bars show the number of papers related to the key topic published in a particular year. Black bars show the number of papers related to “issue prioritization”. White bars show the number of papers related to “pull request prioritization”.

Secondly, we clustered the distribution of topics by year of publication (Figs 5 and 6). The dynamics of growth for the key topics underlying this study are roughly the same. This suggests that the scientific community is equally interested in both topics. As we noted above, this demonstrates their close interrelation.

**Fig 7:**
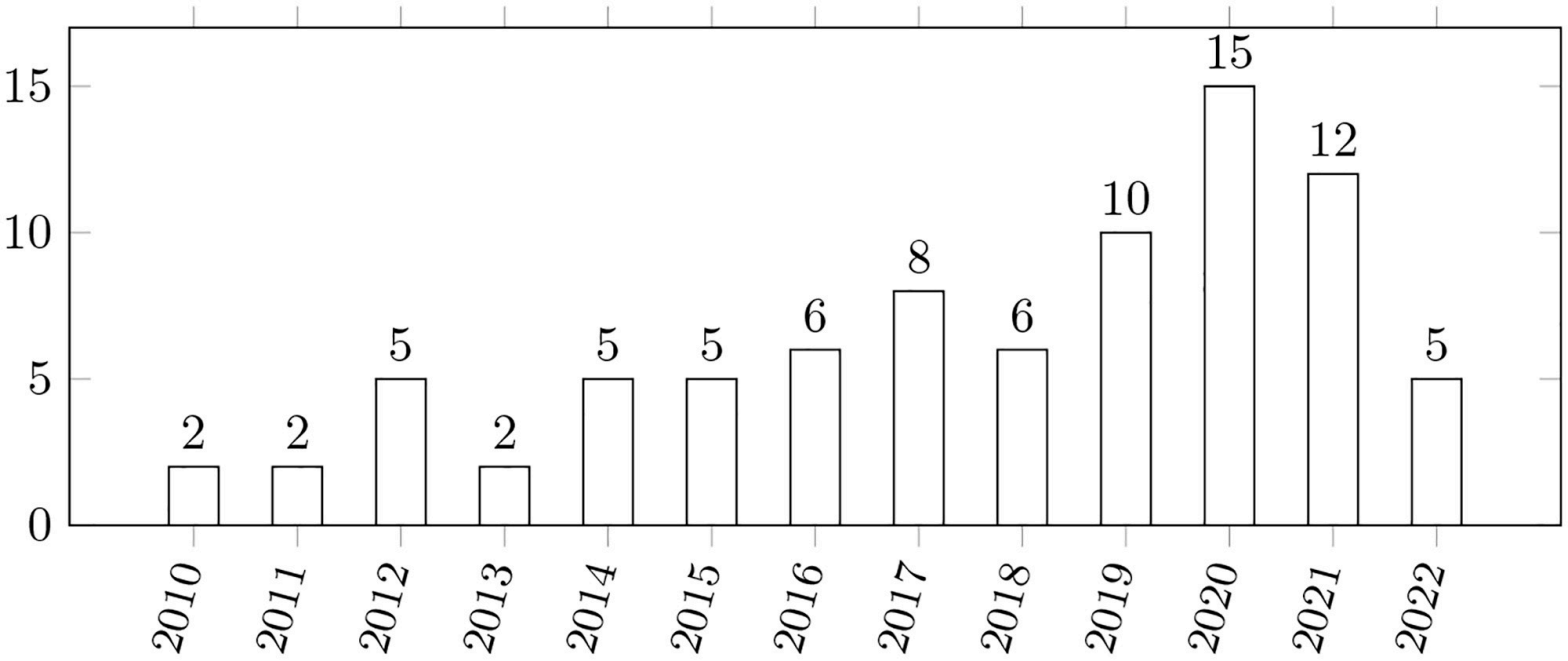
Papers distribution by years. The bars show the number of papers published on the topic between 2010 and 2021. No papers we found for the period 2006–2009. 2006 was the starting year for our SLR IC3.

Thirdly, building and expanding on this classification, we clustered the papers we selected by the year of publication. Fig 7 shows our results. The same information is presented in Table 5, where it is aggregated and visualized over a 4-years period.

**Table 5:** Papers distribution over a 4-years period. The table shows the number (column “Quantity”) and percentage (column “Percentage”) of papers for the specified period (column “Years”).

Years	Quantity	Percentage
2010–2014	16	19.3
2015–2018	25	30.1
2019–2022	42	50.6

Data from Table 5 suggests that the topics of our SLR are becoming the focus of many researchers worldwide (about 50% of the papers included in our reading log were produced in the last four years). We can also observe that the number of papers on these topics has grown at least two times over the last four years. This can be (presumably) explained by the widespread adoption of new techniques in ML, which was probably determined by an increased interest in Artificial Intelligence (AI). To verify this hypothesis, we found the correlation between these two topics by calculating the relevant Pearson coefficient [50], which is given by:

$$\begin{array}{c} r=\frac{\sum \left(x_{i}-\bar{x}\right)\left(y_{i}-\bar{y}\right)}{\sqrt{\sum {\left(x_{i}-\bar{x}\right)}^{2}{\left(y_{i}-\bar{y}\right)}^{2}}}, \end{array}$$ (1)

where *x*_*i*_ is the number of papers published on the keyword “artificial intelligence” counted using the Scopus query (artificial AND intelligence), and *y*_*i*_ is the number of papers relevant for this SLR, for the i-th year in the period 2010–2022.

The Pearson correlation coefficient is 0.9 with a *p*-value of 6.4*e* − 06. This confirms our assumption that there is a significant synergy between the growth in the number of ML tools and their application to our problem domain.

**Fig 8:**
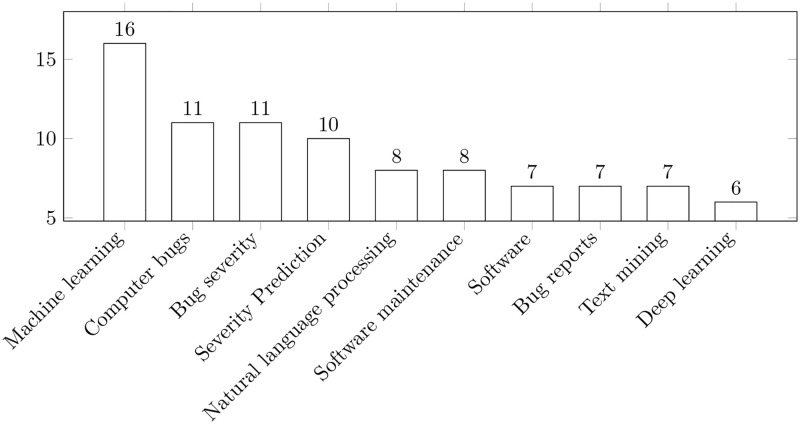
Tags distribution among the papers included in the final reading log. The bars show the number of papers related to a specific tag. Note: several tags can be assigned to one paper.

Fourthly, we also collected some statistics related to the tags used in the papers we included in the final reading log. Information about this point is presented in Fig 8.

We note that the information presented in Fig 8 can be used to:

- add more substance to the conclusions related to algorithm distribution we made in Further Clustering;
- validate the relevance and the significance of our selection (the papers included in the final reading log);
- characterize the most popular “subtopics” investigated by researchers worldwide in the selected domain.

### Further clustering

**Fig 9:**
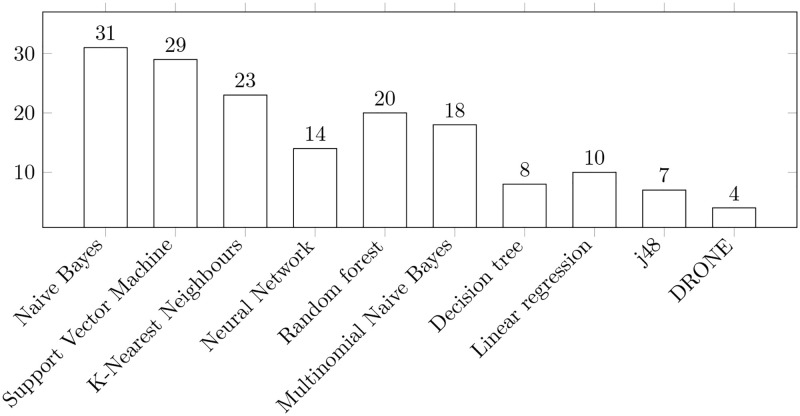
Algorithms used in the papers included in the final reading log. The bars show the number of papers in which the specified algorithms were considered. Note: several algorithms can be considered in one paper.

We next proceed to further cluster our results and we do so along three dimensions: *a)* algorithms, *b)* datasets, and *c)* metrics. Fig 9 shows the algorithms used for training the models. Naive Bayes [51] is the most popular method among the models observed in the papers we reviewed.

**Fig 10:**
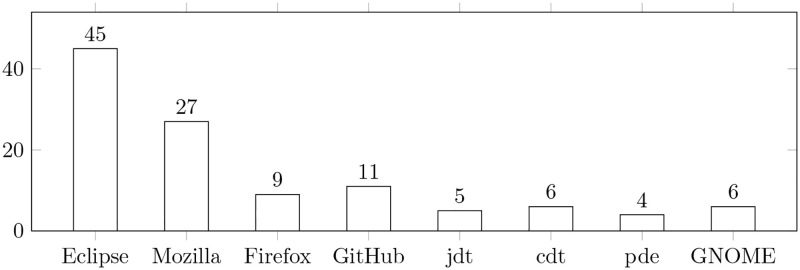
Distribution of datasets. The bars show the number of papers in which the specified algorithms were considered. Note: several datasets could be considered in one paper.

In addition, we also clustered the datasets used in the papers included in the final reading log. The most often used datasets are presented in Fig 10.

Fig 10 shows the datasets most frequently used, which account for 48.7% of all datasets. The remaining datasets, accounting for 51.3% of the total, have been used only once, for example, bug repository of hdfs, etc. It is also worth noting that a single dataset can be found in many articles. The total number of dataset occurrences is calculated based on this important observation.

**Fig 11:**
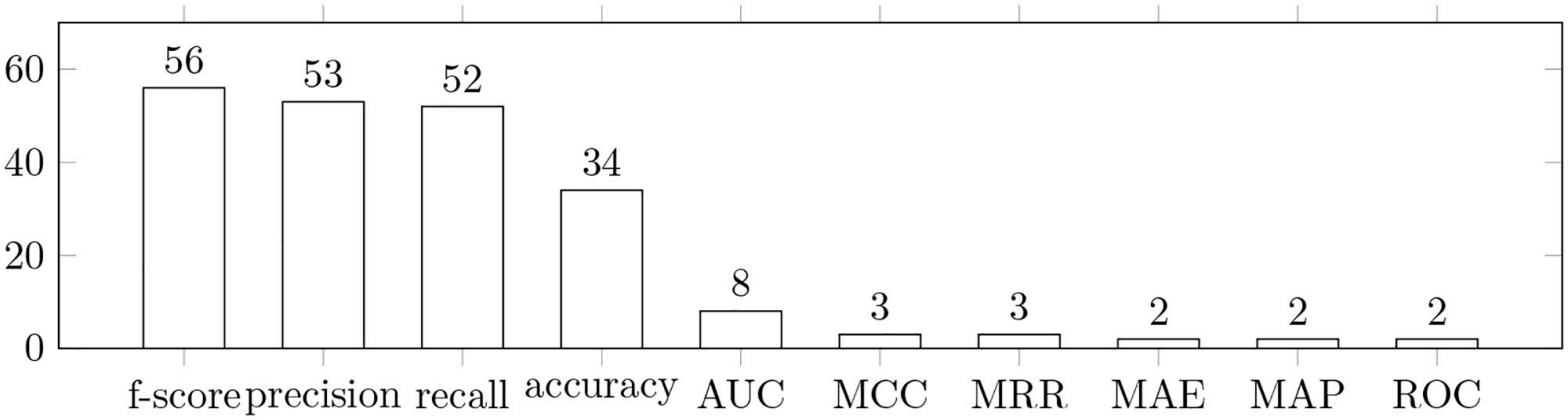
Metrics used in the papers included in the final reading log. The bars show the number of papers in which the specified metrics were used. Note: several metrics could be used in one paper. Acronyms used: AUC—area under the ROC curve, MCC—Matthews Correlation Coefficient, MRR—mean reciprocal rank, MAE—mean absolute error, MAP—mean average precision, ROC—receiver operating characteristic curve.

Finally, we collected statistics about the metrics used in the papers we included in our reading log (Fig 11). We did not plot the metrics reported once. Nevertheless, we believe that such metrics are important because they might be used to create a comprehensive overview of their usage, which can be instrumental in evaluating the effectiveness of task prioritization models. These metrics include: average percentage of faults detected (APFD), normalized discounted cumulative gain (NDCG), mean squared error (MSE), Cohen’s kappa coefficient, nearest false negatives, nearest false positives, adjusted r squared, prediction time, training time, and robustness.

We note that some papers may contain multiple metrics, which might be jointly used to assess and more comprehensively evaluate the quality of a model. The f-score, as shown in Fig 11, is the most commonly used metric in the papers we reviewed.

## Discussion

In this section, we contextualize and critically discuss the data presented in Results, while also highlighting their significance and relevance for the field.

### RQ1. What are the existing approaches for automatic task ranking in software development?

**Fig 12:**
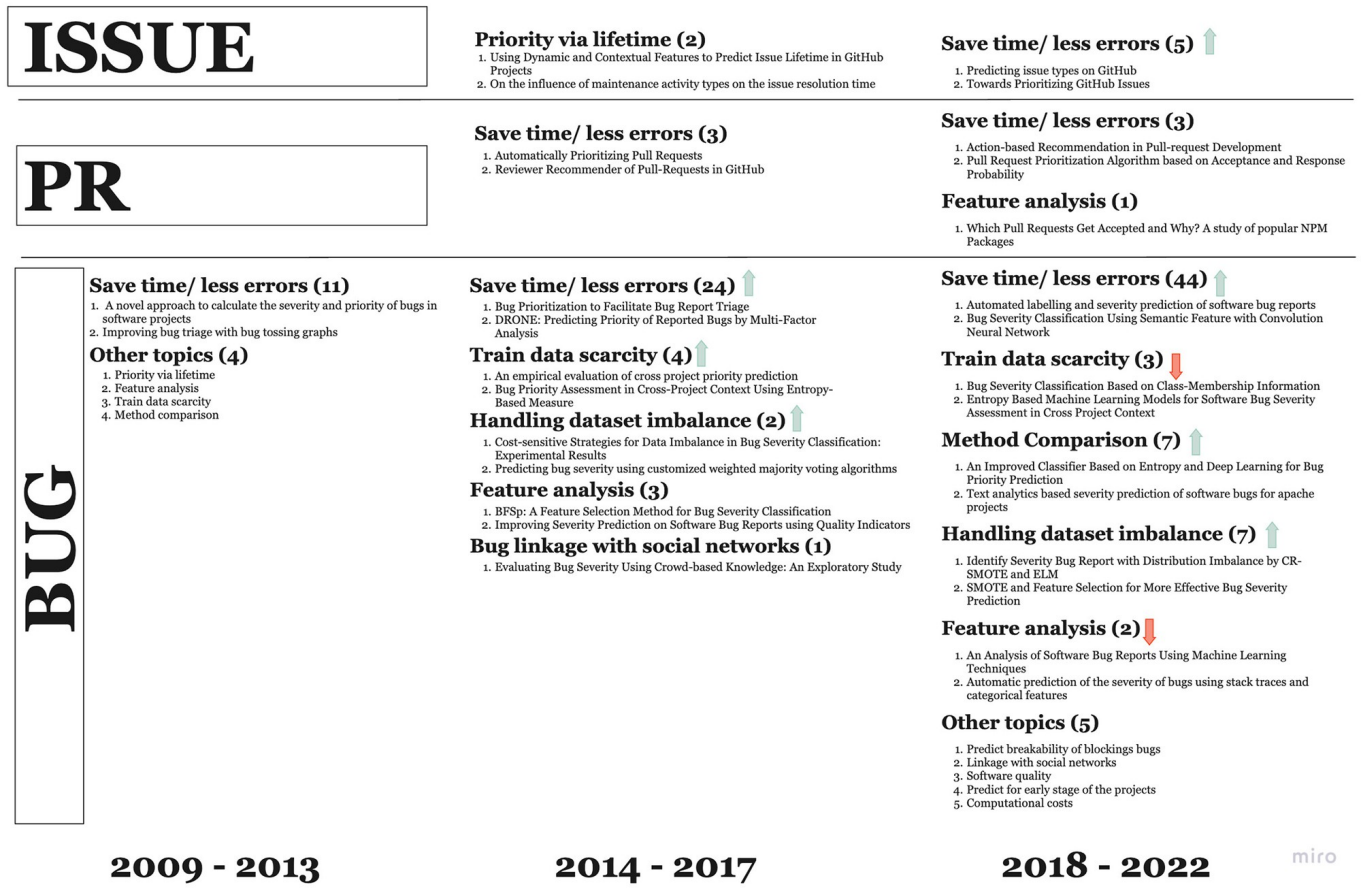
Dynamics of task prioritization research over time. The graph illustrates the chronological development of approaches to task prioritization from 2009 to 2022, specifically with respect to bugs, issues, and pull requests (PR). Each subtopic is denoted by bold text, followed by the total number of associated works. The figure is divided into three vertical fragments, each representing one of the subtopics. Green and red arrows indicate an increase or decrease in the number of publications, respectively.

As discussed in the Introduction, task prioritization can be divided into 3 subtopics: issues prioritization, PRs prioritization, and bugs prioritization. In this study, we treat these topics as independent issues and discuss them below in order of appearance in the literature. Bugs prioritization originated first, and it involves prioritizing the bugs based on their severity and impact on the software system [52]. Issue prioritization is the process of selecting and ranking issues based on factors such as their importance and urgency [53]. Finally, Pull Request prioritization is the process of selecting and ranking pull requests based on their impact on the software system and their relationship with other pull requests [54]. Overall trends in the task prioritization field are shown in Fig 12.

### Bug prioritization

In this work, we understand bugs as reports by users and developers about program components that do not function properly. The collection of attributes used to describe the reports is usually determined by the platform on which the report was created. The platform used to detect bugs in most cases is Bugzilla (see https://www.bugzilla.org/). In 2004, one of the first studies by Cubranic and Murphy [5] on bug prioritization was conducted. Although this paper has been highly influential in the literature, we did not include this study in our main log because it failed to satisfy one of our inclusion criteria (namely, the third criterion).

However, since 2004, which is the year in which this paper was published, the field of bug prioritization boomed, giving raise to many profitable investigations on: prioritization on imbalanced datasets, prioritization in case of scarce datasets, and analyses concerning relevant features in datasets. Each of these areas deals with separate problems inherent to the field of bug prioritization.

**Fig 13:**
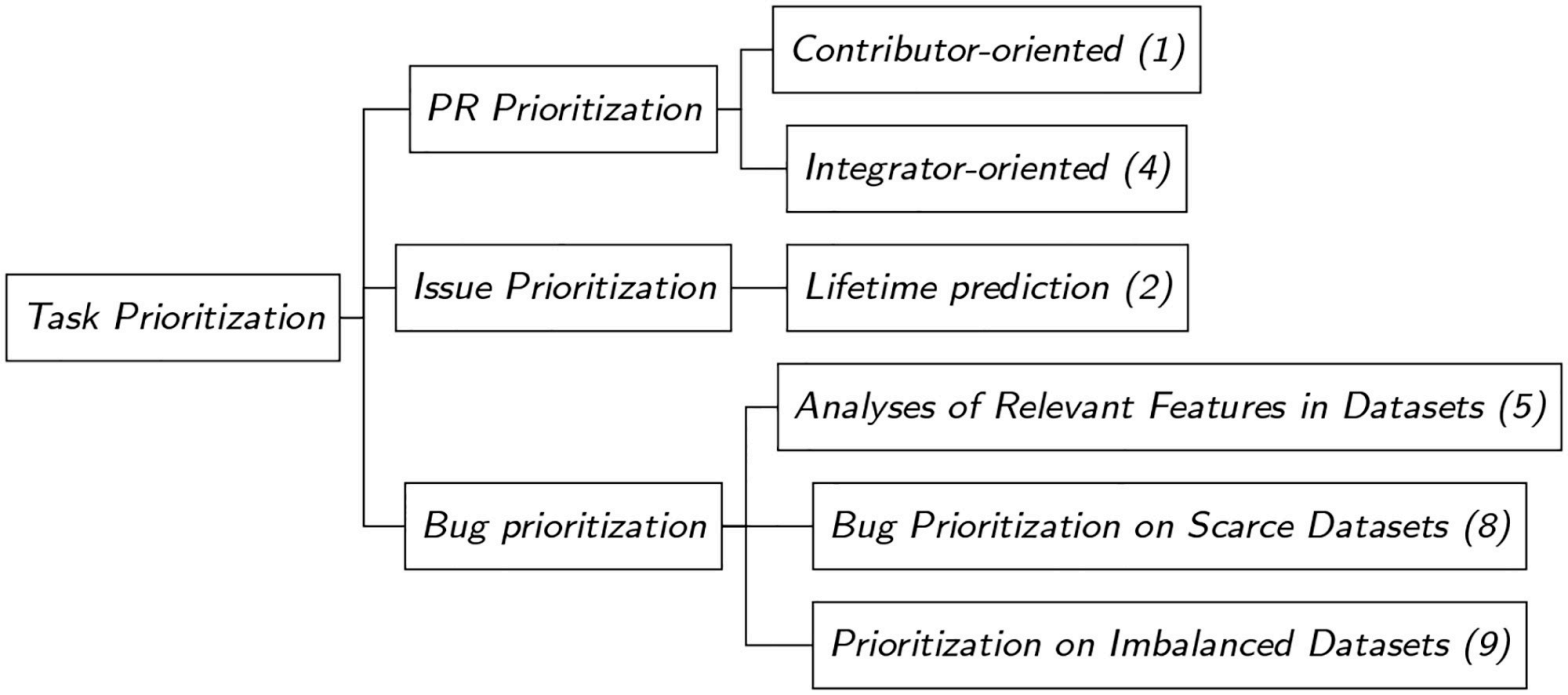
Branches of task prioritization. The numbers in brackets indicate the number of publications found for each branch.

Since we were unable to find precise causal relationships in the development of each of these directions, we describe them below on the basis of their popularity. The popularity of each direction is hereby determined by the number of scientific articles we found to be related to that specific direction. Fig 13 provides information about the popularity of each direction.

#### Prioritization on imbalance datasets

According to Fig 13, the most popular subtopic within bug prioritization is prioritization on imbalanced datasets. We believe that the popularity of this area is determined by the problem it subtends.

In brief, machine learning systems trained on imbalanced data will only perform well on samples with a dominant label. A dataset with a high number of low-priority bugs, for example, is more likely to classify subsequent bugs, even those with a high priority, as low priority. One of the researchers who also noted the importance of balancing the dataset is Thabtah [55]. Having explained the possible reasons for the popularity of this topic, we next move on to analyze its general structure as well as some of the most representative works we found that relate to it.

In light of the data we gathered, we can divide bug prioritization on imbalance datasets into two categories, based on the specific (machine learning) techniques used: those that use one predictor and those that use several predictors (what is known as the ensemble approach).

An example of work belonging to the former category is the work of Singha and Rossi [56]. The authors of this work used a modified version of Support Vector Machine (SVM) to weight classes based on the inverse occurrence of class frequencies. The results suggest that the model provides better prediction quality than standard SVM. Another example of the approach predicated by the former category is the work of Guo et al. [57]. In this study, the authors used Extreme Learning Machine (ELM) as a predictor. Several oversampling strategies were also tested.

The findings of this study are interesting for the purpose of this study because they indicate that the suggested approach can effectively balance an imbalanced dataset, which can contribute to increase the accuracy of bug prioritization.

Methods using a single predictor to deal with bug prioritization on imbalance datasets, as previously indicated, are not the only ones available. An example of work using multiple predictors (hence belonging to the latter category above-mentioned) is the work of Awad et al. [58].

The authors of this work proposed using the so-called ensemble method, in which each category of bug has its own predictor plus an additional general predictor for any type of bug. The topic of ensemble methods was described in more detail by Optiz and Maclin [59]. The peculiarity of the method is that any machine learning technique can be used as a predictor; the authors of the paper tested several techniques (such as Nave Bayes Multinomial (NBM), Random Forest (RF), and SVM). They also evaluated their proposed approach, which used both textual-based and non-textual datasets. Results showed that the proposed method can be successfully used to improve classical methods; however, this could be done only in the presence of a textual dataset.

#### Bug prioritization on scarce datasets

A second subtopic we found within bug prioritization is prioritization in case of scarce data. Research in this area typically attempts to formulate methods capable of showing consistent and accurate results despite the availability of a relatively small amount of training data. The first work we found for bug prioritization in case of scarce data is the work of Sharma et al. [11], which was published in 2012. Several machine learning techniques, like SVM, Naive Bayes (NB), Neural Networks (NN), K-Nearest Neighbours (KNN), were tested to ascertain the best suitable and most accurate among them all. The authors showed that overall SVM and NN produce better results.

It is nevertheless worth noting that M. Sharma is the primary contributor to the topic, having published 5 of the 8 papers we found. We note that in this research group repeatedly utilized the same set of machine learning techniques (such as SVM, NB, NN, and KNN) [11, 60, 61]. We, therefore, acknowledge that this may lead to biased conclusions.

We also note that the techniques listed above are not all the techniques that are currently applied, used, or tested in the literature. For example, Zhang et al. [62] proposed using ELM, while Hernández-González et al. [63] used the Expectation Maximization (EM) Algorithm.

#### Analyses of relevant features in datasets

This brings us to the discussion of the last subtopic within bug prioritization: analyses of relevant features in datasets. The goal of researchers in this field is to identify a set of features within a dataset that will yield the highest accuracy for a model trained on such data.

Although the topic is well-researched, there is still no consensus on the optimal set of attributes to be used. For example, the first publication in the domain by Alenezi and Banitaan [64] indicates that meta-data attributes are more relevant than textual description features. Sharmin et al. [65] also investigate the significance of features; however, they only compare two fields (text description and text conclusion).

Another perspective on the relevance and significance of features/attributes is offered by Sabor et al. [66]. The authors of this article proposed using stack traces as well as attributes, that were discussed by Alenezi and Banitaan [64]. More recently, a few works explored new ways for supplementing datasets with social-media information, for example, the work of Zhang et al. [67].

In light of the evidence reviewed above, we believe that the wide range of techniques and opinions developed in the literature thus far makes the task of identifying optimal qualities considerably challenging. Hence, we fear we are not in a position to make any specific recommendation with respect to this subtopic.

### Issue prioritization

An issue (see https://docs.github.com/en/issues) is an object that describes the work and the prerequisites for completing it. Any member of the open-source community can create an issue in order to enhance any given product. Issues in software development are typically found in platforms like GitHub, GitLab, or Bitbucket. Because of the novelty of these platforms, the subject has received little attention from the research community.

The main approach for dealing with issue prioritization has been that of predicting the lifetime of the issue itself. This approach was initially discussed by Murgia et al. [53]. The authors of this paper also investigate the impact of different types of developers’ activities (such as maintenance type, adding a new feature, refactoring, etc) on issue resolution time (or issue lifetime). These activities are often represented with labels. The results of this study show that fixing defects and implementing/improving new features is more effective and typically less time-consuming than other activities (such as testing or documenting).

The idea of using labels to represent activities also inspired other authors, such as Kikas et al. [68]. Subsequent work by Kallis et al. [69] confirmed the potential of this research direction and analyzed the relationship between static/dynamic features and issues’ lifetime.

Static features are those that remain consistent over time (for example, the number of issues created by the issue submitter in the three months before opening the issue). Dynamic features on the contrary are those features that change depending on when an observation is made (for example, if we look at the number of comments on an issue, we can see how it changes over time).

Another work that attempts to resolve issues prioritization by utilizing the concept of issue-lifetime prediction is the work of Dhasade et al. [70]. The authors of this work continued to use both static and dynamic attributes. They also expanded the previously developed approach by including in the model (and subsequently testing within it) various other hyperparameters (such as time and hotness). The changes implemented in the model by these researchers made the model more flexible and therefore capable of being adjusted to the needs of different teams.

As previously stated, the majority of articles predict the priority based on the expected issue lifetime. A slightly different strategy is however demonstrated by Kallis et al. [69], where labels are used by the authors to assist developers in the organization of their work (hence prioritizing their tasks). The method developed in this study can correctly and reliably anticipate one of three labels: bug, enhancement, or question.

### PR prioritization

We cannot fully understand task prioritization if we do not discuss the third category that falls within it; namely, PR Prioritization. Research on PR Prioritization may be divided into two sub-topics, as shown in Fig 13: integrator-oriented research and contributor-oriented research. An integrator is someone who is in charge of reviewing PRs, whereas a contributor is someone who creates PRs.

In the former category (integrator-oriented research), we may include the works of [54, 71–73]. Van der Veen [71] offered a tool for prioritizing PRs based on static and dynamic attributes. This type of approach is quite similar to the work of Dhasade et al. [70]. In fact, Dhasade et al. [70] were inspired by this work and used it, as we have seen above, as a conceptual palette for their investigation.

A study by Yu et al. [54] proposes another approach to improving PR prioritization. The approach revolves around the idea of recommending appropriate reviewers to PRs. A description of the PR and a comment-network are two of the most crucial features used in this model. The comment network is a graph that is constructed based on the developers’ shared interests. Results from this study show that the method is capable of obtaining a 71 percent precision in predicting the appropriate reviewer.

Another method, that has similar goals, is discussed by Yu et al. [72]. The researchers developed an approach that is intended to aid in the prioritization of PRs, by forecasting their latency (i.e., evaluation time). To make such a prediction, the researchers took into consideration numerous socio-technical parameters (such as project age, team size, and total CI). The findings demonstrated that the length of the dialogue (the number of comments under the PR) had a substantial impact on its latency.

With respect to the latter category we introduced above (contributor-oriented research), we only found one relevant study by Azeem et al. [74]. In this study, the authors not only investigated the impact of each individual variable on the probability of a PR being merged, but they also formulated and developed a model capable of automatically estimating such a probability.

To obtain these results, the researchers used the XGBoost algorithm and over 50 different attributes. The mean average precision of their model for the first five recommended PRs was 95.3%, hovered at 89.6% for the first ten PRs, and eventually decreased to 79.6% for the first twenty PRs. The results show that the technique outperformed the baseline model presented by Gousios et al. [75] at all levels (for the first five, the first ten, and the first twenty PRs).

### RQ2. Which methods are used in automatic task ranking models and approaches and how is their effectiveness assessed?

As we noted in the previous section, giving an exact definition of a task can be quite challenging, at least in software development. In our research, we found that the same approaches are usually employed to solve prioritization tasks for each of those different attributions. In other words, the observations we made for one meaning of the term invariably apply to the others. We speculate that the reason for this might be the presence of some sort of common fields or attributes between all these different meanings.

On these grounds and in light of the data presented in Fig 9, we can conclude that Naive Bayes is the most frequently used technique for solving the problem of predicting bug severity and priority.

In this context, it is essential to note that, despite the growing popularity of neural network approaches in other areas of computer science, we did not entirely observe the same popularity in the studies we selected. We believe that the relative unpopularity of neural networks and deep neural networks [76] might be caused by the relatively small size of the dataset. Even though neural networks are very powerful tools, they require a lot of training to process data properly, which was not always possible, for different reasons, in the papers we reviewed.

As we pointed out in Fig 11, the most used metrics for assessing the effectiveness of models are: f-score, precision, recall, and accuracy. This may indicate that the skewness of the datasets was relatively low [77]; however, closer scrutiny [60, 64, 78] reveals that this is not the case. Only nine studies employ the f-score as the only assessment metric. We can infer that in most cases additional metrics such as precision and recall are used in combination with the f-score to provide a complete and fair characterization of the model’s quality.

**Fig 14:**
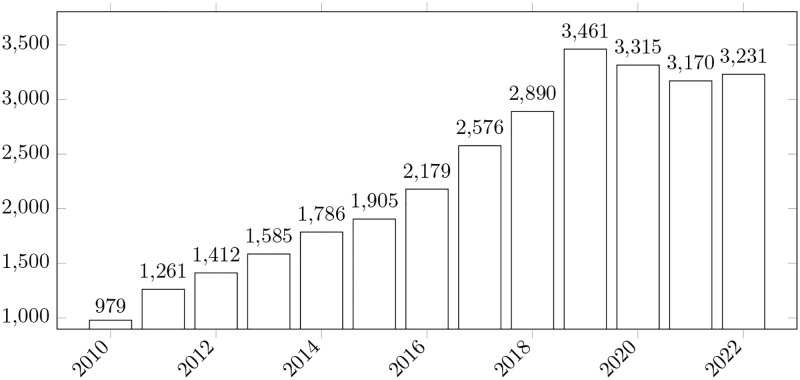
Papers distribution according to the query “recommender system” in scopus. The bars show the number of papers on the topic published between 2010 and 2022.

Based on the results shown in Further Clustering we can make another important observation. Among the most popular metrics observed in our review, we noticed that there were not any of those typically used for recommender systems [79, 80]. Even though the question of task priority has a recommender nature, i.e., we want to know “what are the next tasks/features/bugs and how should be solved”; recommender ML techniques are not so popular. We believe that one of the reasons for that is that recommender system approaches [81] have only recently captured researchers’ attention. This speculation is reinforced by the observation concerning the number of papers published under the search query “recommender system” for the period 2010–2022 (data gathered from Scopus, Fig 14). The development of recommender systems can be a profitable way to guide existing works and the orientation of recommender systems can be used to gauge and support effective decision-making. This means that a model trained in this fashion cares about the ordering of output variables. So, if we have multiple entities and need only a small subset of the best of them, we most probably need to look at the solutions offered by the recommender system.

### RQ3. What are the most effective and versatile models for automatic task ranking developed so far?

Giving a proper answer to the research questions proved to be more challenging due to the high variability of datasets and metrics used, and it may also depend upon the output variables selected (e.g. datasets). If we want to obtain reliable results, we should therefore compare results obtained on the same dataset [82]. Eclipse was the dataset of our choice. We chose Eclipse because it’s the most popular dataset, according to the findings we presented in our results section. We must also note that if the authors made a comparison and/or had multiple models in one of their works, we decided to consider only one model, the one with better performance and the highest results. The result of our comparisons is given in Tables 6 and 7.

**Table 6:** Comparison of methods predicting priority. Priority levels are from Bugzilla [47]. The performance is described as precision/recall/f-score with the best results highlighted in bold. All data are shown as percentages.

Priority levels	Fang et al. [83]	Tian et al. [12]	Kanwal and Maqbool [84]	Pushpalatha et al. [85]	Choudhary and Singh [86]
p1	17 / 35 / 22	0 / 0 / 0	29 /43 / -	**100** / **97** / **98**	74 / 49 / 59
p2	19 / 32 / 24	0 / 0 / 0	29 / 84 / -	**86** / **85** / **85**	74 / 64 / 68
p3	89 / 67 / 77	88 / **100** / 94	**97** / 53 / -	75 / 87 / 80	94 / 98 / **96**
p4	11 / **30** / 16	0 / 0 / 0	37 / 24 / -	48 / 28 / **35**	**52** / 25 / 34
p5	15 / 38 / 21	0 / 0 / 0	16 / 46 / -	**67** / 67 / 67	60 / **85** / **70**

**Table 7:** Comparison of methods predicting severity. Severity levels are from Bugzilla [46]. The performance is described as precision/recall/f-score with the best results highlighted in bold. All data are shown as percentages.

Severity levels	Zhang et al. [87]	Tian et al. [88]	Hamdy and El-Laithy [89]	Sharmin et al. [65]	Pundir et al. [90]	Zhang et al. [91]	Kukkar et al. [92]
blocker	64/78/78	25/27/26	28/26/27	-/-/15	**95**/**83**/**89**	31/25/27	80/75/78
critical	**86**/**91**/**89**	28/30/29	29/26/28	-/-/25	79/69/73	28/30/29	82/78/80
major	87/**96**/**91**	58/58/58	46/62/53	-/-/53	**95**/86/90	60/48/53	80/76/79
minor	97/**96**/97	42/38/40	39/30/34	-/-/31	**100**/95/**97**	43/45/44	80/77/79
trivial	78/88/**82**	28/25/27	42/15/22	-/-/29	68/**100**/81	19/42/26	**83**/79/81

Table 6 shows a comparison between models with respect to their capacity for predicting bugs’ priority. We note that even though the number of papers using the same dataset is much higher, the comparison table has only five elements. That is because some papers either used different levels of priorities and metrics or gave only graphical information, so no accurate value for the metric could be gauged.

As Table 6 shows, the work by Pushpalatha et al. [85] has the highest amount of highlighted cells. This makes it the best approach concerning the Eclipse dataset.

Table 7 shows a severity-wise comparison among all the approaches reviewed.

We note that the highest score is attributed to the approach proposed by [87], while the second-highest result is achieved by the model proposed by [90]. This paper, based on the Naive Bayes ML algorithm, is interesting because it formulated a method capable of ensuring the most accurate result for the blocker bug, which is one of the most severe types of bugs typically found. We also note that works by [87] demonstrated better outcomes for critical bugs.

## Critical review of our research question

Because the analysis we conducted above showed a limited number of research articles related to task prioritization in software development, this prompted us to make several assumptions and partially expand the scope of our study. Since the word “task” can refer to multiple concepts, we decided to consider this word in its most general meaning. This allowed us to gather more articles for our analysis. However, the fact that we only gathered a relatively limited number of scientific articles related to prioritization may indicate a significant gap in the research field. On the one hand, the presence of this gap may be taken as a sign of the relevance and novelty of this study for the research field. On the other hand, our findings raise several significant and pressing concerns. For example, why has so little research been conducted in this area? What are the pitfalls in task prioritization research? Why there was no demand for such a system? While we do not have a clear answer to all these questions, we can nevertheless assert that this SLR highlighted the need for more research in this area (task prioritization in software development) while also forming a solid basis for future progress in the field.

### Analysis of RQ1: What are the existing approaches for automatic task ranking in software development?

We can make several important observations about the results we obtained. Firstly, earlier work dealt mainly with the problem of “bug” prioritization, which, albeit useful, is neither exhaustive nor comprehensive. That is so because we are interested in a broader understanding of task prioritization.

Secondly, only recently (especially in the last 5 years, as shown in Fig 12) researchers began to pay attention to the concept of “pull requests” prioritization and “issues” prioritization, which substantially expanded on original research conducted in the prioritization of Software Development metrics. As we discussed earlier on in this paper, this may well result in substantial growth of the literature in the near future, as it was the case with “bug prioritization” in the past.

### Analysis of RQ2: Which methods are used in automatic task ranking models and approaches and how is their effectiveness assessed?

The number of methods described in this SLR for task prioritization is rather limited. The most popular method we observed is Naive Bayes. This method is important because it provides the most accurate result for the blocker bug. We also analyzed several different metrics found in the papers we reviewed, the most popular of which are: *a)* f-score, *b)* precision, *c)* recall, and *d)* accuracy.

Across the whole set of metrics, we found in the papers we reviewed for this SLR, CPU-costs related metrics are the rarest, which means that the question of computational costs has not been a priority. This may signal a new potential future research direction for task prioritization.

Based on the results presented above, we can argue that the lack of metrics commonly used in recommender systems represents an interesting research gap in the field, which is also shown in [93]. Our explanation for the existence of such a gap lies in the observation that there are still very few studies on recommender systems [81]. This is due, presumably, to the fact that recommender systems only recently attracted researchers’ attention.

### Analysis of RQ3: What are the most effective and versatile models for automatic task ranking developed so far?

As we have shown above, the quality of task prioritization in software development has improved over the years as new and more accurate estimation methods have been deployed [87]. However, when it comes to prioritizing “pull requests” and “issues,” there are a lot of conflating strategies and ideas about what to prioritize [50]. Unfortunately, we have to admit that there isn’t a standardized or universally agreed approach for prioritizing such issues. Because of that, the only way to compare the approaches currently available is to fully reproduce and compare them on the same dataset and on the same set of metrics. This, however, is an extremely complicated task. Nevertheless, developing new works along this research direction may open up new vistas of vital importance for further progress in the field.

### A synoptic summary

The brief synoptic summary of our results with respect to each of the research questions tackled in this study is the following:

**RQ1** The number of articles that consider the problem of automatic task prioritization is still fairly small.
**RQ2** Only a few articles, among those reviewed, assessed models in terms of time or CPU costs. Also, there is a sensitive lack of metrics used to analyze recommender systems.
**RQ3** TSo far, there is no standardized approach or universal agreement on defining prioritization strategies for “pull requests” and “issues.” Presumably, this is because datasets are not marked up, meaning that there are no labels on data samples (see https://www.ibm.com/cloud/learn/data-labeling).

## Limitations, threats to validity, and review assessment

### Limitations

We start this subsection by briefly reviewing some of the obstacles that may have prevented an objective review.

In this study, we used five different databases, namely, *Google Scholar, Microsoft Academic, ScienceDirect, IEEE Xplore, and ACM*. A skeptical reader may point out that we should have used more databases (such as *Springer Link, Web of Science, Scopus, ProQuest, Academic OneFile*). We certainly are aware of the existence of many more databases (besides those we selected) and could even agree that a larger set of databases might have broadened the scope of our work and increased the diversity of our searches. However, we picked those databases most commonly used by software engineers worldwide and that are known to aggregate the largest possible variety of papers. So, even though the list of databases used to perform our searches could have been ameliorated, we are pretty confident that our searches were scientifically sound.

Also, one may see as perhaps problematic the fact that we did not use any kind of grey literature in this work. Although there is a tendency to advocate for the usage of multivocal approaches (such as grey literature) in software engineering, we believe that such practice (given the dubious nature of such literature) should be limited to cases where there is a sensible lack of secondary sources, which was not our case.

Finally, one may rightly claim that in picking only works written in English, we somehow constrained and severely limited the scope and breadth of this research. Cross-cultural issues are emerging as vitally important in ensuring universalism in science. We agree with the importance of adding multicultural perspectives and even under-represented works in any study; however, most of the literature in the field is in English, and all the best journals only accept submissions in English. We, therefore, deem that the requirement we adopted in this SLR concerning language is pretty standard for the field and relatively unproblematic. Nevertheless, we note that our team of researchers is culturally very diverse, as it includes people from four different continents.

### Threats to validity

In this subsection, we discuss a series of biases that might have affected the development and production of our review.

- **Bias towards Primary Sources**—SLRs are usually performed on secondary sources. This is done to maximize objectivity. In this work, we uniquely relied on secondary sources; hence we avoided this potential bias.
- **Selection Bias**—A major risk involved in any SLR is what we may call “selective outcome reporting” or “selection bias.” This typically occurs when the authors present only a selection of outcomes and/or results based on their statistical significance. We note that our reading log consists of only peer-reviewed, high-quality papers. The papers, as noted above, were published by world-leading publishers (such as Springer, Elsevier, ACM, and IEEE Xplore). In addition, we selected papers (methodologies, datasets, and metrics) from several journals as well as from reputable conference proceedings. This ensured a variety of levels of analysis and experimental protocols.
- **Bias in Synthesis**—To avoid this type of bias, which can be considered as an extension of the Selection Bias above-mentioned, we carefully assessed our methodological protocol and -by extension- our findings. Thus, all the researchers involved in this study actively and consistently participated in monitoring each other’s activity to maximize objectivity and minimize mistakes (such as this bias).

### Review assessment

Finally, we want to reflect on the overall quality of our work. To do so, we formulated—inspired by Kitchenham [94], a set of questions, which we critically applied to our results and findings. The questions we formulated and the answers we gave to them follow below.

- **Are the inclusion / exclusion criteria objective and reasonable?** Following the best norms in our discipline, we formulated—before conducting our searches—a set of inclusion and exclusion criteria, which we subsequently applied to finalize the reading log. The criteria we formulated are congruent with those generally used in the field and are obviously relevant to the topic of our work.
- **Has there been a quality review?** We developed a metric to assess the papers’ quality (Quality Assessment). We proved that the quality of the papers we included in the log was relatively high. Thus, we can confidently assert that the results that informed our work were scientifically sound and academically grounded.
- **Were the basic data / studies adequately described?** We build a comprehensive literature log. The log consisted of all the relevant information we extracted from the papers we analyzed. This allowed us to process our data transparently and comprehensively. It also ensured the replicability of our findings, which is another key trait of any SLR.

## Conclusion

This SLR investigated the problem of task prioritization in software development and focused on: *a)* identifying existing approaches for automatic task prioritization (RQ1), *b)* further investigating methods and metrics for task prioritization as developed in the literature (RQ2), and *c)* analyzing the effectiveness and reliability of these methods and metrics (RQ3).

Concerning RQ1, our results showed that earlier work mainly dealt with bug prioritization, and more recent work has expanded to consider prioritizing pull requests and issues. We speculate that this may lead to a substantial growth of literature in the future. RQ2 revealed that the most popular method used for task prioritization is Naive Bayes, while the most popular metrics used (in descending order) are f-score, precision, recall, and accuracy. However, there is a lack of metrics used in recommender systems, which may indicate a potential direction for future research. RQ3 showed that the quality of task prioritization in software development has improved over time; however, there is still a sensible lack of standardized approaches for prioritizing pull requests and issues.

In light of these findings, we can assert that this SLR contributed to broadening the field of research on task prioritization in software development, while also providing a solid basis for future research. Our goal in the mid-term is to develop an empirical study based on the topic of this SLR. Our aim in such a study would be to find a practical way to implement the findings of this review. To this extent, we shall consider whether it could be possible to develop algorithms for predicting task prioritization in a project using ML methods. This may well lead to novel AI-based management strategies, which could improve people’s well-being at work as well as foster moral and social good.

Nevertheless, IT practitioners should be cognizant of the relatively scarce amount of research conducted on task prioritization to date. They should also be aware of the absence of established methods for prioritizing pull requests and issues. They should therefore use the results of this SLR as a springboard for further explorations aimed at the development of such methods and tools.

## Supporting information

S1 Table PRISMA 2020 checklist. Template is taken from: www.prisma-statement.org/documents/PRISMA_2020_checklist.pdf.
(PDF)

S2 Table Quality scores assigned to the papers. (PDF)
