# Supplementary material for: Prioritizing tasks in software development: A systematic literature review
Source: PLoS One. 2023 Apr 6;18(4):e0283838. doi: 10.1371/journal.pone.0283838 (PMC10079059; doi:10.1371/journal.pone.0283838)
Supplement: S1 Table — Template is taken from: www.prisma-statement.org/documents/PRISMA_2020_checklist.pdf. (PDF) [file pone.0283838.s001.pdf]

Section and Topic	Item #	Location
**TITLE**		
Title	1	
**ABSTRACT**		
Abstract	2	Abstract
**INTRODUCTION**		
Objectives	3	Introduction
PaperStructure	4	Introduction
**METHODS**		
Eligibility criteria	5	SLR Protocol Development
Information sources	6	SLR Protocol Development
Search strategy	7	SLR Protocol Development
Selection process	8	SLR Protocol Development
Data collection process	9	SLR Protocol Development
Data items	10	n/a
Study risk of bias assessment	11	n/a
Effect measure	12	n/a
Synthesis methods	13	n/a
Reporting bias as asessment	14	n/a
Certainty assessment	15	n/a
**RESULTS**		
Study selection	16	n/a
Study characteristics	17	Results
Risk of bias in studies	18	n/a
Results of individual studies	19	Limitations, Threats to Validity, and Review Assessment
Results of syntheses	20	Discussion
Reporting biases	21	Limitations, Threats to Validity, and Review Assessment
Certainty of evidence	22	n/a
**DISCUSSION**		
Discussion	23	Discussion
**OTHER INFORMATION**		
Registration and protocol	24	n/a
