# Supplementary material for: Prioritizing tasks in software development: A systematic literature review
Source: PLoS One. 2023 Apr 6;18(4):e0283838. doi: 10.1371/journal.pone.0283838 (PMC10079059; doi:10.1371/journal.pone.0283838)
Supplement: S2 Table — (PDF) [file pone.0283838.s002.pdf]

Reference	QA1	QA2	QA3	QA4	Sum
Veen et al. (2015)	0.0	1.0	1.0	1.0	3.0
Panichella et al. (2021)	0.0	1.0	1.0	0.5	2.5
Azeem, Panichella, et al. (2020)	0.5	1.0	0.5	1.0	3.0
Kikas et al. (2016)	0.0	0.5	0.5	1.0	2.0
Izadi et al. (2022)	0.5	1.0	1.0	1.0	3.5
Zhao et al. (2019)	0.5	1.0	0.0	1.0	2.5
Azeem, Peng, et al. (2020)	1.0	0.0	1.0	0.0	2.0
Dhasade et al. (2020)	1.0	0.5	1.0	1.0	3.5
Kallis et al. (2021)	1.0	0.5	1.0	1.0	3.5
Fazayeli et al. (2019)	0.5	1.0	1.0	1.0	3.5
Huang et al. (2022)	1.0	1.0	0.5	1.0	3.5
Shatnawi and B. Alazzam (2022)	0.5	0.5	1.0	0.0	2.0
G. Yang, T. Zhang, et al. (2014)	1.0	1.0	1.0	1.0	4.0
M. Sharma, Bedi, K. Chaturvedi, et al. (2012)	1.0	1.0	1.0	1.0	4.0
Tunali (2022)	1.0	1.0	1.0	1.0	4.0
Alenezi and Banitaan (2013)	1.0	0.5	0.5	0.0	2.0
Kanwal and Maqbool (2012)	1.0	1.0	1.0	1.0	4.0
Umer, H. Liu, and Sultan (2018)	1.0	1.0	1.0	0.0	3.0
Gökçeoğlu and Sözer (2021)	1.0	1.0	1.0	1.0	4.0
Umer, H. Liu, and Illahi (2020)	1.0	0.0	0.5	1.0	2.5
Bani-Salameh et al. (2021)	0.5	0.5	0.5	0.5	2.0
Fang et al. (2021)	1.0	1.0	0.5	0.5	3.0
Choudhary and S. Singh (2017)	1.0	0.5	1.0	0.0	2.5
Zhou and H. Zhang (2012)	1.0	1.0	0.5	1.0	3.5
Agrawal and Goyal (2021)	1.0	1.0	1.0	0.5	3.5
T. Zhang, J. Chen, et al. (2016)	0.5	0.5	0.0	1.0	2.0
Sabor et al. (2020)	1.0	1.0	0.5	1.0	3.5
G. Sharma et al. (2015)	0.5	0.0	1.0	1.0	2.5
Tan et al. (2020)	1.0	1.0	0.0	0.0	2.0
Kukkar and Mohana (2018)	1.0	1.0	1.0	0.0	3.0
W. Liu et al. (2018)	1.0	0.0	0.5	0.5	2.0
Chauhan and R. Kumar (2019)	1.0	1.0	1.0	0.5	3.5
M. N. Pushpalatha and M. Mrunalini (2021)	0.5	0.0	1.0	1.0	2.5
Gujral et al. (2015)	1.0	1.0	1.0	0.5	3.5
Baarah, Aloqaily, Salah, et al. (2019)	1.0	1.0	0.5	1.0	3.5
C.-Z. Yang et al. (2014)	0.5	1.0	1.0	0.5	3.0
Jin, Dashbalbar, G. Yang, B. Lee, et al. (2016)	0.5	0.5	1.0	0.0	2.0
K. K. Chaturvedi and V.B. Singh (2012b)	1.0	0.0	1.0	0.0	2.0
Hamdy and El-Laithy (2019)	0.5	1.0	1.0	1.0	3.5
Kukkar, Mohana, and Y. Kumar (2020)	0.5	0.5	1.0	1.0	3.0
V. B. Singh et al. (2017)	1.0	1.0	0.5	1.0	3.5
Sharmin et al. (2017)	0.5	0.0	1.0	1.0	2.5
Jin, Dashbalbar, G. Yang, J.-W. Lee, et al. (2016)	0.5	1.0	0.0	1.0	2.5
Y. Yang and X. Chen (2022)	1.0	0.5	1.0	0.5	3.0
G. Yang, Min, et al. (2019)	0.5	1.0	0.5	0.0	2.0
Tian, Lo, Xia, et al. (2014)	1.0	1.0	1.0	1.0	4.0
Lamkanfi, Demeyer, Giger, et al. (2010)	1.0	1.0	0.5	1.0	3.5
Lamkanfi, Demeyer, Soetens, et al. (2011)	1.0	0.5	1.0	0.5	3.0
K. K. Chaturvedi and V. B. Singh (2012a)	0.5	1.0	0.5	0.5	2.5
Arokiam and Bradbury (2020)	1.0	1.0	0.5	0.0	2.5
T. Zhang, G. Yang, et al. (2015)	1.0	1.0	1.0	1.0	4.0
N. K.-S. Roy and Rossi (2014)	1.0	1.0	1.0	1.0	4.0
Guo et al. (2019)	1.0	1.0	0.5	0.5	3.0
Tian, Lo, and Sun (2013)	0.5	1.0	0.5	1.0	3.0
Kumari and V. B. Singh (2019)	1.0	0.0	0.5	0.5	2.0
Tian, Lo, and Sun (2012)	0.0	1.0	0.0	1.0	2.0
Ramay et al. (2019)	1.0	1.0	0.0	0.5	2.5
Gupta et al. (2022)	1.0	1.0	0.5	0.5	3.0
M N Pushpalatha and Mrunalini (2016)	0.5	1.0	0.5	1.0	3.0
Malhotra et al. (2021)	1.0	1.0	1.0	1.0	4.0
Kim and G. Yang (2022)	0.5	1.0	0.5	1.0	3.0
Alia et al. (2018)	1.0	1.0	1.0	1.0	4.0
Otoom et al. (2016)	1.0	0.5	0.0	0.5	2.0
Tahir et al. (2021)	1.0	0.5	0.5	0.5	2.5
Dao and C.-Z. Yang (2021)	1.0	0.0	0.5	1.0	2.5
Awad et al. (2017)	1.0	0.5	0.5	0.5	2.5
I. Alazzam et al. (2020)	0.5	1.0	0.5	1.0	3.0
Ahmed et al. (2021)	0.5	1.0	0.5	0.5	2.5
Mondreti and Satish (2020)	0.5	0.0	0.5	1.0	2.0
Iqbal et al. (2020)	1.0	0.5	0.5	0.5	2.5
Y. Zhang et al. (2015)	0.0	0.5	1.0	1.0	2.5
G. Yang, Baek, et al. (2017)	0.5	1.0	0.0	1.0	2.5
Tran et al. (2019)	0.0	1.0	0.0	1.0	2.0
M. Sharma, Kumari, et al. (2020)	0.5	1.0	0.5	1.0	3.0
Baarah, Aloqaily, Zyod, et al. (2021)	0.0	1.0	1.0	0.5	2.5
M. Sharma, Bedi, and V. Singh (2014)	0.5	0.5	1.0	1.0	3.0
Kaur and Jindal (2019)	1.0	0.5	1.0	0.0	2.5
W. Zhang and Challis (2019)	1.0	1.0	1.0	1.0	4.0
M N Pushpalatha and Mrunalini (2016)	0.5	1.0	0.5	1.0	3.0
Kukkar, Mohana, Nayyar, et al. (2019)	0.5	0.0	0.5	1.0	2.0
Kanwal and Maqbool (2010)	1.0	1.0	0.5	1.0	3.5
N. K. S. Roy and Rossi (2017)	1.0	0.5	1.0	0.5	3.5
Kumari, U. K. Singh, et al. (2020)	1.0	0.5	0.0	0.5	2.0
